# Comparison of children with bioinactive growth hormone, small for gestational age, and idiopathic short stature

**DOI:** 10.3389/fendo.2025.1596976

**Published:** 2025-09-11

**Authors:** Esma Kaya Özdemir, Esra Döğer, M. Orhun Çamurdan, Aysun Bideci

**Affiliations:** Gazi University Faculty of Medicine, Department of Child Health and Diseases, Department of Pediatric Endocrinology, Ankara, Türkiye

**Keywords:** bioinactive growth hormone, small for gestational age, idiopathic short stature, recombinant human growth hormone, insulin-like growth factor 1, insulin-like growth factor binding protein-3, growth velocity, height z-score

## Abstract

**Introduction:**

Short stature has many causes, including rare disorders of GH function. Bioinactive growth hormone (GH) refers to a phenotype characterized by immunoreactive but biologically ineffective GH. Importantly, it should not be regarded as a separate treatment but rather as a definable subgroup within the broader population of children receiving recombinant human growth hormone (rhGH) therapy. The aim of this study was to compare the growth response to rhGH among children with bioinactive GH, those born small for gestational age (SGA), and those with idiopathic short stature (ISS).

**Methods:**

In this retrospective, single-center study, we reviewed the medical records of short-statured patients with a height ≤ –2 z-score, a normal peak GH response (≥10 ng/mL) to clonidine or L-dopa stimulation tests, and a history of rhGH treatment. Patients with chronic illness, malnutrition, syndromic or endocrine disorders, diabetes, metabolic disease, anemia, or prior pubertal suppression were excluded. Eligible patients meeting the definitions of bioinactive GH, SGA, or ISS were included.

**Statistical Analysis:**

Data were analyzed with IBM SPSS Statistics 22.0 using parametric and non-parametric tests with Bonferroni correction; significance was set at p < 0.05.

**Results:**

Among 170 patients screened, 109 fulfilled the criteria for analysis (bioinactive GH, n=8; SGA, n=27; ISS, n=74). Baseline Insulin-like Growth Factor 1 *(*IGF-1) and Insulin-like Growth Factor Binding Protein 3 (IGFBP-3) levels were markedly lower in the bioinactive GH group compared with SGA and ISS (p < 0.001). During rhGH therapy, patients with bioinactive GH exhibited the greatest gains in growth velocity and Δ height z-score, despite similar GH doses and a lower proportion of pubertal subjects. By final height, all patients with bioinactive GH achieved normal stature, with most surpassing target height, whereas fewer SGA and ISS patients reached their genetic potential.

**Conclusion:**

Children with bioinactive GH form a biologically distinct and highly treatment-responsive subgroup of non-GHD short stature. Our findings highlight the diagnostic value of IGF-1 generation testing in this context. Future multicenter studies with genetic and bioactivity confirmation are essential to refine diagnostic criteria and establish international guidelines.

## Introduction

Short stature is a common clinical issue in pediatric endocrinology with a diverse range of underlying causes, including both pathological conditions and constitutional factors. Although growth hormone deficiency (GHD) is a well-established and treatable cause of short stature, a subset of children exhibit significant growth failure despite having normal peak growth hormone (GH) levels on stimulation tests. This discrepancy reveals the limitations of current diagnostic approaches and draws attention to underrecognized GH dysfunctions, such as bioinactive GH. Bioinactive GH denotes a phenotype in which circulating GH is immunoreactive but biologically ineffective; it is not a distinct therapeutic intervention but rather an identifiable subgroup among children receiving recombinant human growth hormone (rhGH) (1–10).

Bioinactive GH is a rare but clinically important condition characterized by circulating GH that is immunoreactive but biologically inactive due to structural abnormalities of the GH molecule, often associated with pathogenic variants in the GH1 gene. These abnormal GH isoforms fail to effectively bind or activate the GH receptor, resulting in impaired activation of downstream signaling pathways, including JAK2–STAT5 signaling, which is essential for insulin-like growth factor-1 (IGF-1) production and linear growth. Despite normal GH secretion, affected children typically have significantly reduced serum IGF-1 and Insulin-like Growth Factor Binding Protein 3 (IGFBP-3) levels, along with a poor correlation between GH and IGF-1 concentrations, leading to short stature due to functional impairment of GH action (11–14). Initially described by Kowarski in 1978 (2) and further characterized at the molecular level by Takahashi in the 1990s (3), bioinactive GH remains underdiagnosed, with its true prevalence unknown due to limited awareness and the absence of standardized diagnostic tests. Recent reviews highlight the continued lack of standardized diagnostic tools and emphasize the need for harmonized international diagnostic criteria (15).

Previous studies have consistently shown that children with bioinactive GH respond well to exogenous rhGH therapy, despite normal endogenous GH secretion (15–17). Their growth patterns are largely comparable to those of patients with classical GHD, and in some cases, an even greater response has been observed (17). This paradox arises because exogenously administered rhGH retains full biological activity, effectively compensating for the functional defect of the endogenous hormone. Collectively, these findings indicate that bioinactive GH represents a GH-responsive phenotype with a pathophysiological profile more closely resembling GHD than other non-GHD etiologies of short stature.

Accordingly, the present study compares patients with bioinactive GH to two well-established non-GHD short stature cohorts: small for gestational age (SGA) and idiopathic short stature (ISS) (6, 8). Both SGA and ISS represent heterogeneous populations characterized by normal GH secretion but variable responsiveness to rhGH, thereby providing suitable comparator groups for evaluating the growth potential of bioinactive GH. Children born SGA typically experience intrauterine growth restriction (18–22) and demonstrate highly variable patterns of postnatal catch-up growth, whereas ISS is a diagnosis of exclusion applied to children with unexplained short stature in the absence of identifiable systemic, endocrine, or genetic abnormalities (8).

We hypothesize that children with bioinactive GH—representing a distinct subset of non-GHD short stature patients identified through IGF-1 generation testing—will demonstrate a stronger growth response to rhGH therapy, as measured by annual growth velocity, changes in height z-score, and final height z-score, compared with children diagnosed with SGA or ISS.

## Patients and methods

### Patients

Between January 2000 and January 2020, we conducted a retrospective review of medical records from patients treated with rhGH at our center who achieved a peak GH response ≥10 ng/mL on at least one conventional stimulation test with oral clonidine or L-dopa. Patients were excluded if they had chromosomal abnormalities, syndromic disorders, malnutrition, additional hormonal deficiencies (e.g., thyroxine or cortisol), insulin-dependent diabetes mellitus, bone disorders, chronic or metabolic diseases, anemia, or a history of pubertal suppression therapy. All patients underwent detailed physical examination and anthropometric assessment to rule out skeletal dysplasia; none showed disproportion or other physical findings suggestive of such conditions. At rhGH initiation, all patients had a height ≤ −2 z-score and a body mass index (BMI) > −2 z-score.

### Definitions of study groups

IGF-1 and IGFBP-3 z-scores were calculated using sex- and age-specific reference data by Güven et al. (23). Patients with baseline IGF-1 ≤ −2 z-score underwent an IGF-1 generation test: subcutaneous rhGH 0.1 mg/kg/day for four consecutive days with serum IGF-1 and IGFBP-3 re-measured on day five. Patients demonstrating an increase of ≥40 ng/mL in IGF-1 and ≥400 ng/mL in IGFBP-3 were classified as having bioinactive GH (4). Infants with birth weight and/or length ≤ −2 z-score for gestational age were classified as SGA according to sex-specific standards (24). Patients with a height ≤ −2 z-score based on age- and sex-specific norms and without an identifiable cause were categorized as ISS (8).

### Measurements and procedures

Height was measured with a wall-mounted stadiometer (Seca^®^; Seca GmbH & Co. KG, Hamburg, Germany) with patients barefoot and in a neutral stance. Body weight was measured using a digital scale (Fakir Hausgeräte) with a sensitivity range of 100 g to 150 kg while patients wore light clothing. Serum hormone levels were measured using a chemiluminescent immunoassay (Access 2 Immunoassay System; Beckman Coulter Inc., USA).

From medical records we extracted birth weight (BW), pre-treatment height, weight, BMI, and their z-scores; bone age (BA); IGF-1 and IGFBP-3 levels with corresponding z-scores; height and height z-scores at the first, second, and third years after treatment initiation; growth velocities (GV); and final height with z-score.

GV was defined as the annual height increment in cm/year. Pre-treatment GV was calculated from the 12-month interval immediately preceding rhGH initiation. Height age was defined as the age corresponding to the child’s current height on national reference curves. Final height was defined as height at growth cessation (BA ≥15 years in females and ≥16 years in males or GV <1 cm/year).

BMI was calculated as weight (kg) divided by height (m²). Height and BMI z-scores were assessed using Turkish reference data by Neyzi et al. (25). Target height was calculated as follows: females, [(mother’s height + father’s height − 13)/2]; males, [(mother’s height + father’s height + 13)/2]. Target height z-scores were derived relative to national reference standards Neyzi et al. (25). BA was evaluated using the Greulich–Pyle method.

## Statistical analysis

Data were analyzed using IBM SPSS Statistics Version 22.0 (26). Normality was assessed with the Shapiro–Wilk and Kolmogorov–Smirnov tests, together with evaluation of skewness and kurtosis. Parametric data (mean ± SD) were compared using one-way ANOVA with Bonferroni *post-hoc* tests, while non-parametric data (median, Q1–Q3) were analyzed with the Kruskal–Wallis test, followed by Mann–Whitney U tests with Bonferroni correction when significant. Within-group longitudinal changes were assessed using repeated-measures ANOVA with Greenhouse–Geisser correction for normally distributed variables, followed by Bonferroni-adjusted pairwise comparisons. Time-point multiplicity was controlled using Bonferroni correction across time points. A p-value < 0.05 was considered statistically significant.

Height, growth velocity, and biochemical measurements were included in year-specific analyses only when complete data for that time point were available. Complete-case analyses were performed without imputation, and missingness was assumed to be missing completely at random (MCAR) or missing at random (MAR). The reduction in sample size in later treatment years reflects patients who either discontinued rhGH therapy, transferred follow-up to other institutions, or had incomplete documentation for that interval.

## Results

**Figure 1:**
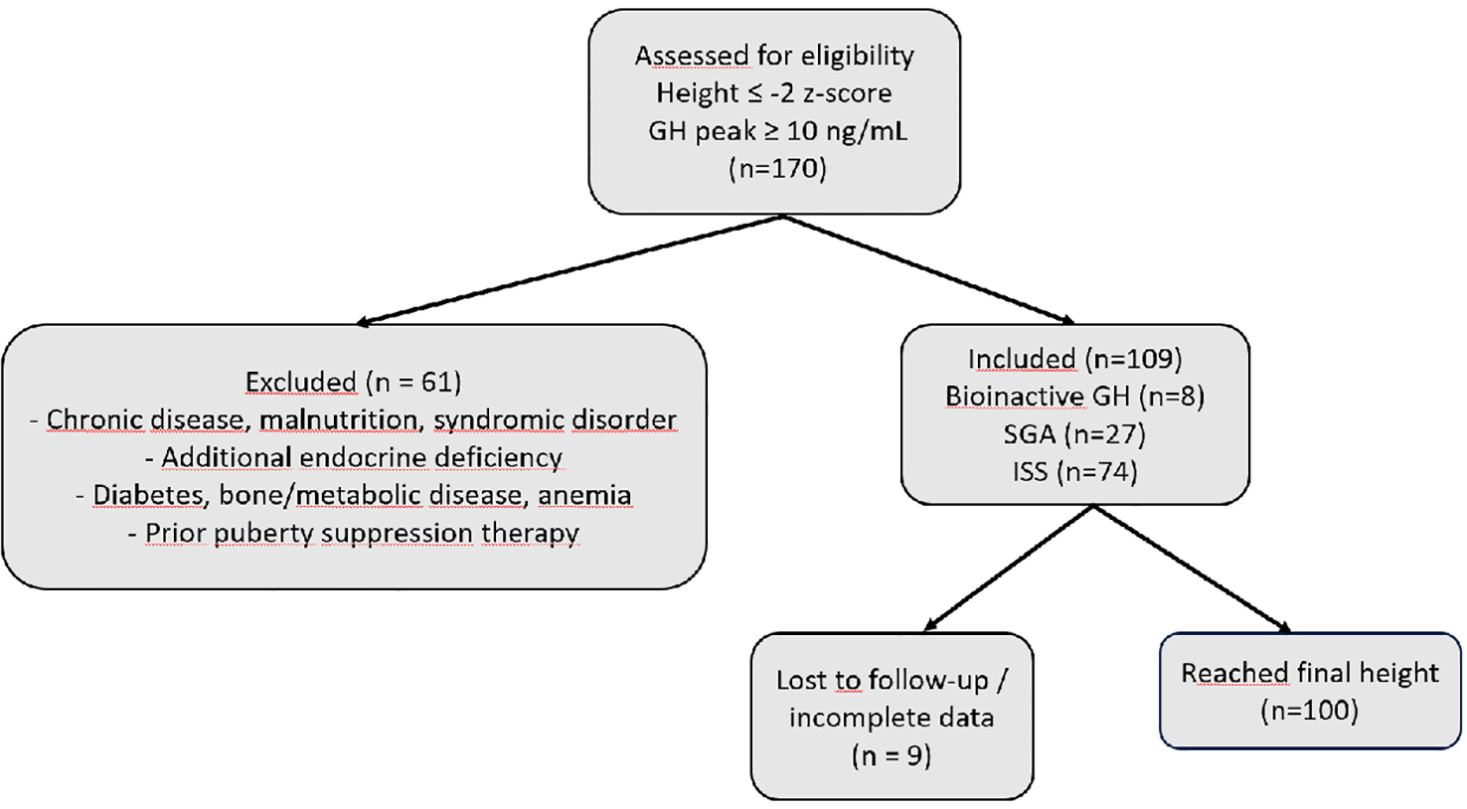
Flow diagram of patient selection and inclusion in the study. Abbreviations: GH, growth hormone; ISS, idiopathic short stature; SGA, small for gestational age; n, sample size; z-score = standard deviation score.

In this study, out of 170 short-statured patients (height ≤ –2 z-score) who demonstrated a normal GH response (≥10 ng/mL) to clonidine or L-dopa stimulation tests and received rhGH treatment at our center, a total of 109 patients classified as bioinactive GH (n = 8), SGA (n = 27), and ISS (n = 74) according to the definitions provided in the Methods section were selected and included in the analysis. The selection of patients is summarized in **Figure 1**.

Baseline characteristics are shown in **Table 1**. Sex distribution was similar descriptively across groups (bioinactive GH: 38% male; SGA: 37% male; ISS: 40% male).

**Table 1:** Baseline anthropometric and parental characteristics of the study groups.

	Bioinactive GH (n=8, mean ± SD)	SGA (n=27, mean ± SD)	ISS (n=74, mean ± SD)	p-value
Male, n (%)	3, 38%	10, 37%	30, 40%	–
BW (g)	3105 ± 349^b^	2148 ± 466^a^	2947 ± 595^b^	p < 0.001
BW z-score	-0.81 ± 0.91^b^	-2.75 ± 0.55^a^	-0.76 ± 0.76^b^	p < 0.001
Pubertal, %	25%	44%	56%	–
Age (years)	10.1 ± 2.75	10.1 ± 3.05	11.3 ± 2.17	0.089
Height age (years)	7.0 ± 2.43	7.5 ± 2.67	8.2 ± 1.87	0.121
BA (years)	8.5 ± 3.48	9.3 ± 2.98	9.9 ± 2.39	0.278
Mother’s height (cm)	150.2 ± 5.92	152.5 ± 5.32	152.7 ± 6.16	0.541
Father’s height (cm)	160.6 ± 3.79^a^	165.9 ± 6.50^b^	168.2 ± 6.80^b^	0.017
TH z-score	-2.28 ± 0.66^a^	-1.69 ± 0.68	-1.49 ± 0.84^b^	0.032

BA, bone age; BW, birth weight; GH, growth hormone; ISS, idiopathic short stature; SGA, small for gestational age; TH, target height; n, sample size; z-score, standard deviation score.

Values are presented as mean ± SD unless otherwise indicated. Superscript letters (a, b) indicate statistically significant differences (p < 0.05); groups sharing the same letter are not significantly different.

BW and BW z-score were significantly lower in the SGA group compared with both the bioinactive GH and ISS groups (p < 0.001). The mean BW was 2148 ± 466 g in the SGA group, 3105 ± 349 g in the bioinactive GH group, and 2947 ± 595 g in the ISS group. The mean BW z-score was –2.75 ± 0.55 in the SGA group, –0.81 ± 0.91 in the bioinactive GH group, and –0.76 ± 0.76 in the ISS group.

At treatment initiation, 25% of the bioinactive GH group, 44% of the SGA group, and 56% of the ISS group were pubertal.

No statistically significant differences were observed among the groups in terms of age, height age, BA, or maternal height. Paternal height differed significantly among groups (p = 0.017), being lowest in the bioinactive GH group (160.6 ± 3.79 cm) compared with SGA (165.9 ± 6.50 cm) and ISS (168.2 ± 6.80 cm). Similarly, target height z-score was significantly lower in the bioinactive GH group (–2.28 ± 0.66) than in the ISS group (–1.49 ± 0.84; p < 0.001).

Biochemical parameters related to the GH-IGF axis before treatment are shown in **Table 2**. All patients in the bioinactive GH group had IGF-1 and IGFBP-3 z-scores below –2 z-scores. Both the absolute levels and z-scores of IGF-1 and IGFBP-3 in this group were markedly lower compared with the SGA and ISS groups (p < 0.001 for all comparisons).

**Table 2:** Baseline IGF-1 and IGFBP-3 levels and z-scores.

	Bioinactive GH (n=8, mean ± SD / median [Q1–Q3])	SGA (n=27, mean ± SD / median [Q1–Q3])	ISS (n=74, mean ± SD / median [Q1–Q3])	p-value
IGF-1 (ng/mL)	41 (33.8-55.8)	157.0 (108.0-235.5)	148.5 (99.5-216.2)	p < 0.001
IGF-1 z-score	-2.48 ± 0.43^a^	-0.79 ± 0.61^b^	-0.76 ± 0.77^b^	p < 0.001
IGFBP-3 (ng/mL)	1360.0 (1147.5-1642.5)	3954.0 (3784.0-4562.5)	3608.5 (3084.8-4440.0)	p < 0.001
IGFBP-3 z-score	-2.40 ± 0.4^a^	-0.28 ± 0.78^b^	-0.65 ± 0.86^b^	p < 0.001

GH, growth hormone; IGF-1, insulin-like growth factor 1; IGFBP-3, IGF-binding protein 3; ISS, idiopathic short stature; SGA, small for gestational age; n, sample size; z-score, standard deviation score.

Values are presented as mean ± SD or as median (Q1–Q3) as appropriate. Superscript letters (a, b) indicate statistically significant differences (p < 0.05).

There was no significant difference in pre-treatment GV among the groups (bioinactive GH: 3.7 ± 0.8 cm/year; SGA: 4.8 ± 1.7 cm/year; ISS: 4.7 ± 1.5 cm/year; p = 0.172).

During the first treatment year, GV increased substantially in all groups, with the highest mean GV observed in the bioinactive GH group (9.1 ± 1.4 cm/year) compared with SGA (8.2 ± 1.9 cm/year) and ISS (8.4 ± 1.9 cm/year; p = 0.504). GH doses were similar across groups (mean 0.032–0.033 mg/kg/day). Approximately half of the bioinactive GH group were pubertal at this stage, compared with 63% in the SGA group and 80% in the ISS group.

In the second year, the bioinactive GH group again showed the highest GV (8.6 ± 2.2 cm/year) relative to SGA (6.7 ± 2.1 cm/year) and ISS (7.5 ± 1.9 cm/year; p = 0.10). GH dose was comparable across groups (p = 0.31), and the proportion of pubertal patients increased in all cohorts, with ISS showing the highest rates.

In the third year, mean GV remained higher in the bioinactive GH group (8.2 ± 2.2 cm/year) than in the SGA (6.4 ± 1.7 cm/year) and ISS (6.9 ± 2.1 cm/year) groups (p = 0.372). GH doses did not differ significantly, and most patients in all groups were pubertal by this stage.

Final height analysis showed a greater total height gain in the bioinactive GH group (40.7 ± 12.5 cm) compared with SGA (28.2 ± 15.4 cm) and ISS (29.2 ± 11.0 cm), although this difference was not reach statistical significance (p = 0.071).

Longitudinal changes in height z-score from the initiation of rhGH therapy to final height are summarized in **Table 3**. At baseline, mean height z-scores were similar across groups (bioinactive GH: –3.14 ± 0.33; SGA: –3.02 ± 0.60; ISS: –2.92 ± 0.62; p = 0.572).

**Table 3:** Longitudinal changes in height z-score across study groups.

	Bioinactive GH (mean ± SD)	SGA (mean ± SD)	ISS (mean ± SD)	p-value
Height z-score 0	-3.14 ± 0.33 (n=8)	-3.02 ± 0.60 (n=27)	-2.92 ± 0.62 (n=74)	0.572
Height z-score 1	-2.33 ± 0.52 (n=8)	-2.48 ± 0.62 (n=27)	-2.59 ± 0.67 (n=74)	0.434
Height z-score 2	-2.14 ± 0.63 (n=5)	-2.14 ± 0.47 (n=18)	-2.28 ± 0.72 (n=55)	0.704
Height z-score 3	-1.96 ± 0.48 (n=4)	-1.88 ± 0.58 (n=11)	-1.95 ± 0.80 (n=30)	0.960
Height z-score F	-1.30 ± 0.77 (n=6)	-2.11 ± 0.96 (n=25)	-1.79 ± 0.95 (n=69)	0.091

Height z-score 0, baseline; Height z-score 1-3, after 1st, 2nd, and 3rd years of rhGH therapy; Height z-score F, final height.

Other abbreviations are as in **Table 1**.

After the first year of treatment, height z-scores improved in all groups (bioinactive GH: –2.33 ± 0.52; SGA: –2.48 ± 0.62; ISS: –2.59 ± 0.67; p = 0.434). Incremental gains were maintained in the second and third treatment years, with no significant differences between groups (p = 0.704 and p = 0.960, respectively).

At final height, mean z-scores reached –1.30 ± 0.77 in the bioinactive GH group, –2.11 ± 0.96 in the SGA group, and –1.79 ± 0.95 in the ISS group (p = 0.091). Within-group analyses confirmed significant improvements in height z-scores over time in all cohorts (p < 0.001). Corresponding GH doses and pubertal distribution are presented in **Table 4**.

**Table 4:** Growth velocity, GH dose, and pubertal status before and during rhGH treatment.

	Bioinactive GH (mean ± SD)	SGA (mean ± SD)	ISS (mean ± SD)	p-value
Pre-treatment GV (cm/year)	3.7 ± 0.8 (n=8)	4.8 ± 1.7 (n=22)	4.7 ± 1.5 (n=66)	0.172
Pubertal status (%)	25%	44%	56%	–
GV, year 1 (cm/year)	9.1 ± 1.4 (n=8)	8.2 ± 1.9 (n=27)	8.4 ± 1.9 (n=74)	0.504
GH dose (mg/kg/day)	0.032	0.033	0.031	0.381
Pubertal, %	50%	63%	80%	–
GV, year 2 (cm/year)	8.6 ± 2.2 (n=5)	6.7 ± 2.1 (n=19)	7.5 ± 1.9 (n=55)	0.103
GH dose (mg/kg/day)	0.033	0.038	0.035	0.310
Pubertal status (%)	60%	61%	93%	–
GV, year 3 (cm/year)	8.2 ± 2.2 (n=4)	6.4 ± 1.7 (n=11)	6.9 ± 2.1 (n=30)	0.372
GH dose (mg/kg/day)	0.034	0.042	0.039	0.138
Pubertal status (%)	75%	91%	97%	–
Total height gain (cm)	40.7 ± 12.5 (n=6)	28.2 ± 15.4 (n=25)	29.2 ± 11 (n=69)	0.071

GV, growth velocity. Other abbreviations are as in **Table 1**.

Values are presented as mean ± SD unless otherwise indicated. Sample sizes per year are indicated in parentheses where they differ from baseline.

**Figure 2:**
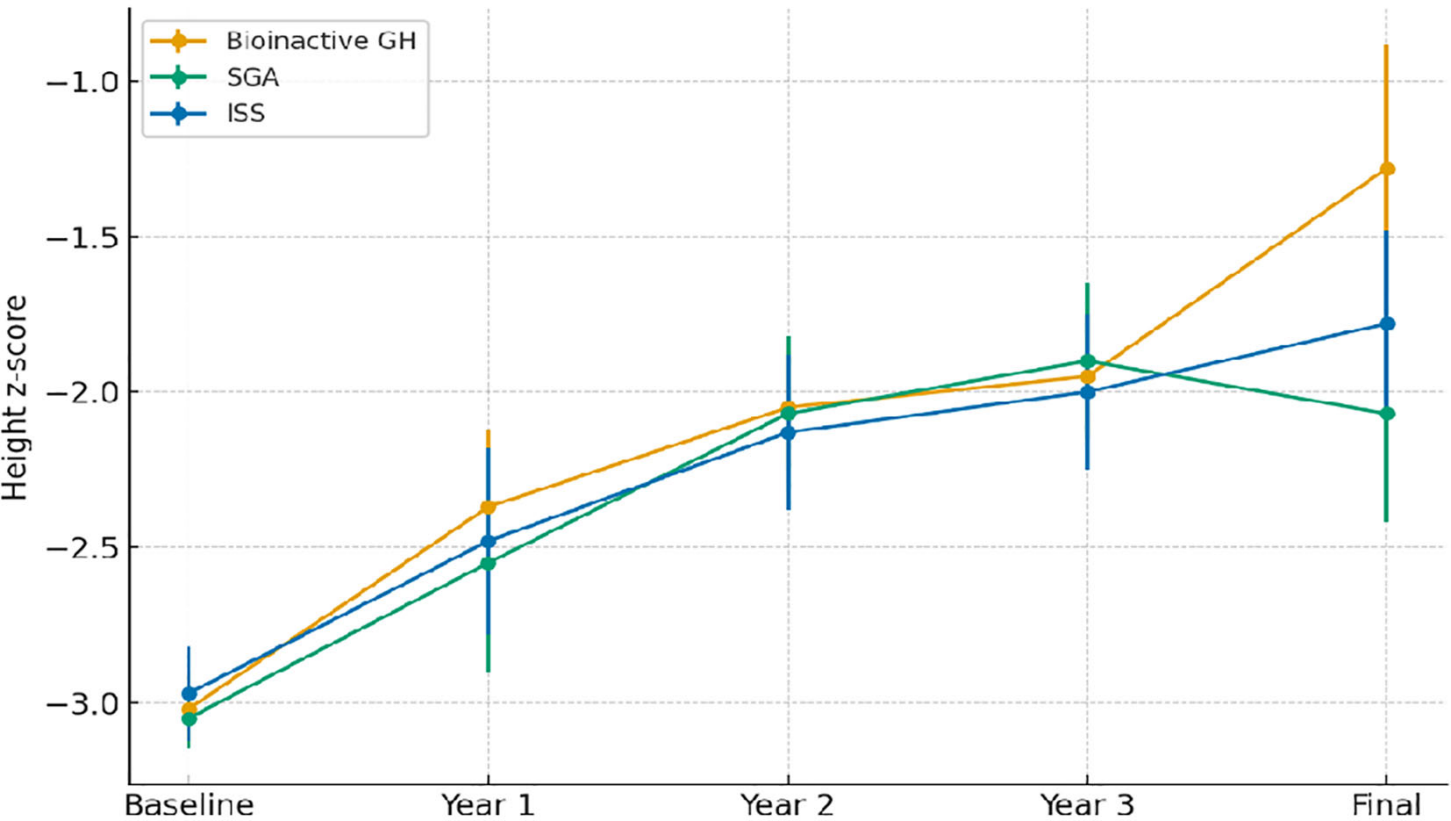
Longitudinal changes in height z-scores by study group. Abbreviations are as in **Table 1**.

**Figure 2** illustrates longitudinal changes in height z-scores across the three study groups.

Yearly and cumulative changes in height z-score (Δ height z-score), together with corresponding GH doses and pubertal distribution, are presented in **Table 5**.

**Table 5:** Annual and cumulative Δ height z-score.

	Bioinactive GH (mean ± SD)	SGA (mean ± SD)	ISS (mean ± SD)	p-value
Δ height z-score 1	0.81 ± 0.37^a^ (n=8)	0.54 ± 0.48 (n=27)	0.33 ± 0.38^b^ (n=74)	0.024
Δ height z-score 2	0.48 ± 0.25 (n=5)	0.26 ± 0.26 (n=18)	0.38 ± 0.38 (n=55)	0.347
Δ height z-score 3	0.41 ± 0.07 (n=4)	0.34 ± 0.36 (n=11)	0.43 ± 0.28 (n=30)	0.681
Δ height z-score 0-3	1.39 ± 0.21 (n=4)	1.05 ± 0.66 (n=11)	1.05 ± 0.72 (n=30)	0.152
Δ height z-score 0–F	1.86 ± 0.85 (n=6)	0.85 ± 0.96 (n=25)	1.21 ± 1.02 (n=69)	0.073

Δ height z-score = change in height z-score; Δ height z-score 1-3 = annual year-to-year changes; Δ height z-score 0-3 = cumulative change over the first 3 years; Δ height z-score 0–F = change from baseline to final height.

Other abbreviations are as in **Table 1**.

At the end of the first year, the mean Δ height z-score in the bioinactive GH group was 0.81 ± 0.37, higher than both the ISS group (0.33 ± 0.38, p = 0.024) and the SGA group (0.54 ± 0.48). GH dosing was comparable across groups, and the stronger response in the bioinactive GH group occurred despite a lower proportion of pubertal patients.

In the second year, the Δ height z-score remained higher in the bioinactive GH group (0.48 ± 0.25) compared with SGA (0.26 ± 0.26) and ISS (0.38 ± 0.38), though differences were not significant (p = 0.347). In the third year, Δ height z-scores were similar among groups.

Cumulatively over the first three years, the bioinactive GH group showed the highest total Δ height z-score (1.39 ± 0.21), compared with 1.05 in both SGA and ISS (p = 0.152). From baseline to final height, Δ height z-score was greatest in the bioinactive GH group (1.86 ± 0.85) versus SGA (0.85 ± 0.96) and ISS (1.21 ± 1.02), approaching statistical significance (p = 0.073).

**Figure 3:**
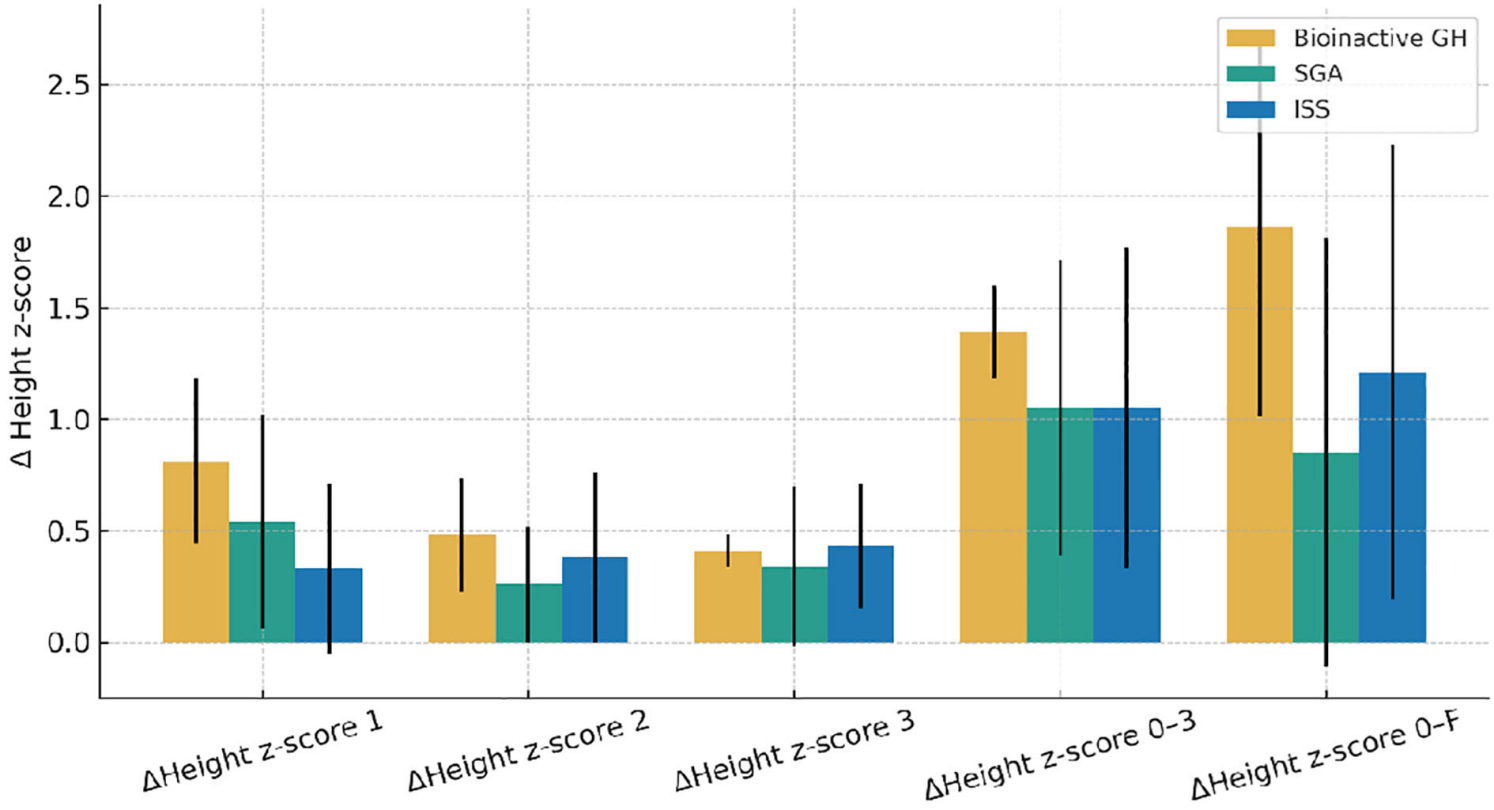
Annual and cumulative changes in height z-scores by group. Abbreviations are as in **Table 1** and **Table 5**.

**Figure 3** shows the annual and cumulative Δ height z-score in the three groups—bioinactive GH, SGA, and ISS—following rhGH therapy.

Among the 100 patients who reached final height, 57 (57%) achieved a normal height (height > −2 z-score). In the bioinactive GH group, all 6 patients (100%) reached normal height, and 5 (83%) achieved their target height. In the SGA group, 11 out of 25 patients (44%) reached normal height and 9 (36%) reached target height, whereas in the ISS group, 40 out of 69 patients (58%) attained normal height and 29 (42%) reached target height.

Comparisons of target height z-scores and observed height z-scores at baseline, the third year of treatment, and final height are presented in **Table 6**. At baseline, the difference between target and observed height z-scores (Δ height z-score_target–observed) was significantly higher in all groups, indicating a marked initial height deficit relative to genetic potential. The largest discrepancy was observed in the ISS group (1.41 ± 0.90), followed by the SGA (1.30 ± 0.70) and bioinactive GH (0.94 ± 0.77) groups.

**Table 6:** Comparison of target and observed height z-scores at baseline, year 3, and final height.

Group	Baseline Δ (target–observed) height z-score	Year 3 Δ (target–observed) height z-score	Final Δ (target-observed) height z-score	p-value
Bioinactive GH	0.94 ± 0.77^a^	-0.45 ± 0.83^b^	-0.92 ± 0.87^b^	p < 0.001
SGA	1.30 ± 0.70^a^	0.25 ± 0.69^b^	-0.09 ± 0.84^b^	p < 0.001
ISS	1.41 ± 0.90^a^	0.35 ± 1.02^b^	-0.02 ± 0.91^b^	p < 0.001

Δ height z-score = difference between target height z-score and observed height z-score.

Other abbreviations are as in **Table 1**.

After three years of rhGH therapy, all groups demonstrated significant improvement, with the gap between target and observed height z-scores narrowing considerably. In the bioinactive GH group, this difference became negative by year 3 (−0.45 ± 0.83) and further declined to −0.92 ± 0.87 at final height, indicating that most patients exceeded or approached their genetic potential. The SGA and ISS groups also showed progressive narrowing of the target–observed gap, reaching near parity by final height (−0.09 ± 0.84 and −0.02 ± 0.91, respectively), though less pronounced than in the bioinactive GH group.

Within-group changes over time were statistically significant in all three groups (p < 0.001).

## Discussion

This study provides one of the most detailed comparative analyses to date of growth outcomes in children with bioinactive GH versus two major non-GH-deficient short stature populations: SGA and ISS. All participants exhibited normal peak GH responses on stimulation testing yet demonstrated distinct patterns of responsiveness to rhGH therapy. Children classified as having bioinactive GH based on IGF-1 generation test results demonstrated a more pronounced and consistent growth response than both the SGA and ISS groups, reflected in annual growth velocity, increases in height z-score, and attainment of higher final height z-scores. These findings highlight the clinical relevance of bioinactive GH as a distinct phenotype and reinforce the diagnostic utility of IGF-1 generation testing in selected patients with unexplained short stature.

A notable strength of this investigation is the direct, protocol-consistent comparison of three clinically meaningful subgroups treated in a single tertiary center, ensuring standardized dosing, monitoring, and anthropometric assessments. This methodological uniformity minimizes variability related to treatment approach or measurement techniques, thereby increasing the reliability of between-group comparisons. The consistent superiority of growth outcomes in the bioinactive GH group further supports the validity of this classification, despite the absence of confirmatory molecular or bioactivity assays.

### Limitations and diagnostic challenges

The retrospective design and single-center setting of this study inherently constrain causal inference and limit external validity. The small number of patients with bioinactive GH (n = 8) reflects both the rarity of this phenotype and its probable underdiagnosis, resulting in reduced statistical power and wide confidence intervals of the estimated effects. While the low prevalence of bioinactive GH partly explains the limited cohort size, assembling a larger sample through coordinated multicenter collaboration would enhance statistical robustness, narrow confidence intervals, permit adequately powered subgroup analyses, and facilitate exploration of genotype–phenotype correlations.

Furthermore, the exclusive inclusion of Turkish patients treated at a single tertiary center introduces potential selection bias and may limit generalizability to populations with different genetic architectures, environmental exposures, or healthcare system structures. Prior studies have demonstrated that ethnic and environmental variability can significantly influence baseline growth patterns, pubertal timing, and responsiveness to rhGH therapy (27, 28), supporting the need for cautious extrapolation of the present findings to other contexts (4, 14, 29). Addressing these limitations through prospective, ethnically diverse, multicenter cohorts will be essential for establishing validated, widely applicable diagnostic and therapeutic recommendations for bioinactive GH.

The classification of bioinactive GH in this study was based solely on clinical and biochemical criteria, particularly IGF-1 generation testing, without molecular or *in vitro* bioactivity confirmation. GH immunoassays detect immunoreactive hormone but cannot differentiate structurally altered or functionally impaired isoforms, a discrepancy recognized for decades. Immunofunctional assays that quantify GH capable of bivalent receptor binding can enrich for biologically competent fractions, but their limited availability and imperfect correlation with *in vivo* signaling restrict clinical utility. Cell-based bioassays, such as the Nb2 rat lymphoma proliferation assay and Ba/F3–STAT5 luciferase reporter systems, differ in specificity and sensitivity; although the latter can detect markedly reduced bioactivity, both may fail to capture intermediate phenotypes and lack cross-platform comparability (12). Moreover, while several pathogenic GH1 variants (e.g., R77C, D112G) are known to produce biologically inactive GH, many patients with reduced GH bioactivity have no GH1 mutations, underscoring phenotypic heterogeneity and the need for multimodal confirmation (4, 30, 31). These diagnostic constraints may result in misclassification, potentially underestimating or overestimating the prevalence and treatment responsiveness of bioinactive GH in broader clinical populations.

Emerging evidence supports integrating next-generation sequencing (NGS) into the diagnostic work-up for growth disorders to identify GH1 and other relevant gene variants. However, interpretation of variants of uncertain significance (VUS) remains a challenge. Incorporating standardized immunofunctional and bioactivity assays alongside genetic testing would improve diagnostic certainty, refine patient stratification, and facilitate international comparability. Future prospective, multicenter studies should integrate these advanced modalities with phenotypic data to establish validated, widely applicable diagnostic algorithms for bioinactive GH, ultimately enabling more tailored therapeutic strategies (32).

### Superior rhGH responsiveness in bioinactive GH

Children with bioinactive GH demonstrated markedly greater gains in height z-score and growth velocity throughout the treatment period compared with SGA and ISS peers, despite receiving similar GH doses and having a lower proportion of pubertal subjects during early therapy. Remarkably, all patients in this group achieved final heights within the normal range, and most exceeded their mid-parental height expectations, an outcome uncommon in other non-GHD populations. These findings align with reports by Binder et al. and Pagani et al., which documented striking catch-up growth in patients with reduced GH bioactivity but intact GH secretion (15, 16).

Mechanistically, bioinactive GH is characterized by immunoreactive hormone that is structurally or functionally impaired, often due to missense mutations or post-translational modifications in GH1 (3, 4, 7). This defective hormone fails to effectively activate GH receptors or downstream JAK2–STAT5 signaling, leading to low IGF-1 and IGFBP-3 levels despite normal circulating GH. Administration of exogenous rhGH—unaffected by these molecular defects—restores signaling pathways and drives robust linear growth. The substantial increases in IGF-1 and IGFBP-3 levels observed in our cohort after IGF-1 generation testing are consistent with this mechanism (33).

### Distinction from SGA and ISS

While SGA and ISS patients also showed height improvements, these were generally less pronounced and more variable. SGA is frequently associated with intrauterine growth restriction and possible epigenetic modifications, placental insufficiency, or fetal programming that impair GH signaling at the receptor or post-receptor level (34, 35). Even with adequate GH secretion, residual effects of prenatal growth restriction can blunt catch-up growth, and responsiveness to rhGH often plateaus after 2–3 years in some cohorts (36, 37).

ISS represents a diagnosis of exclusion, encompassing etiologies from polygenic short stature to unrecognized syndromes and subtle endocrine dysfunctions (38–41). The heterogeneity of ISS explains the wide variation in rhGH responsiveness, as reflected by the large standard deviations in Δ height z-score in our ISS cohort. While ISS patients in our study achieved continued gains over time—possibly aided by IGF-1-guided dose adjustments and increased pubertal prevalence—fewer attained their target height compared with the bioinactive GH group. Collectively, these findings align with recent literature indicating that the rhGH response in SGA and ISS is largely determined by underlying pathophysiological mechanisms rather than GH secretion itself (17, 42).

### Role of pubertal timing and height potential

Pubertal onset is a major determinant of growth rate and can confound the evaluation of rhGH efficacy. In our cohort, although the proportion of pubertal patients in the bioinactive GH group was lower during the first three years of treatment, greater increases in Δ height z-scores were achieved compared with the other groups. This suggests that their superior response is driven by treatment-specific factors rather than puberty-related acceleration. Furthermore, despite starting with the lowest target height z-score, these patients surpassed their predicted genetic height by final assessment—contrasting with SGA and ISS patients, who generally did not exceed mid-parental expectations. This reinforces the notion that bioinactive GH represents a biologically distinct and highly treatment-responsive subgroup.

### Diagnostic and clinical implications

Bioinactive GH is rare and diagnostically challenging, as standard stimulation protocols assess immunoreactivity but not bioactivity, potentially leading to missed diagnoses. While GH1 sequencing, Nb2 proliferation, and Ba/F3–STAT5 assays can evaluate bioactivity, these remain largely confined to research settings and are not widely available (12–14). In this context, IGF-1 generation testing offers a practical, accessible diagnostic option when GH secretion is normal but IGF-1 is unexpectedly low.

Our results support incorporating IGF-1 generation testing into diagnostic algorithms for children with unexplained short stature, low baseline IGF-1, and normal stimulation test results. A robust rise in IGF-1 and IGFBP-3 following short-term rhGH administration should prompt consideration of bioinactive GH and can justify initiating therapy even without classical GH deficiency confirmation. Although genetic and bioactivity assays remain important confirmatory tools, IGF-1 generation testing is a valuable decision-making aid when these are unavailable.

## Conclusion

Children with bioinactive GH form a biologically distinct and highly treatment-responsive subgroup of non-GHD short stature. Our findings highlight the diagnostic value of IGF-1 generation testing in this context. Future prospective, multicenter studies integrating genetic and bioactivity confirmation are essential to refine diagnostic criteria and establish internationally applicable guidelines for clinical management.

## Data Availability

The original contributions presented in the study are included in the article and its supplementary material. Further inquiries can be directed to the corresponding author.
